# Correction: Deciphering the LRRK code: LRRK1 and LRRK2 phosphorylate distinct Rab proteins and are regulated by diverse mechanisms

**DOI:** 10.1042/BCJ20200937_COR

**Published:** 2025-03-26

**Keywords:** kinase, leucine rich repeat kinase, phosphorylation, Rab GTPase

It has come to the attention of the authors of the article “Deciphering the LRRK code: LRRK1 and LRRK2 phosphorylate distinct Rab proteins and are regulated by diverse mechanisms” (DOI: 10.1042/BCJ20200937) that there was a repetition of blots representing GFP (LRRK1) in the upper and lower panels of Figure 9. This was an unintentional error made during the assembly of the Figure, whereby the same blot for GFP (LRRK1) was selected for the two panels. The Figure has been corrected to display the blots for GFP (LRRK1) in both panels, consistent with the raw data (Licor scanned images).

**Figure 9:**
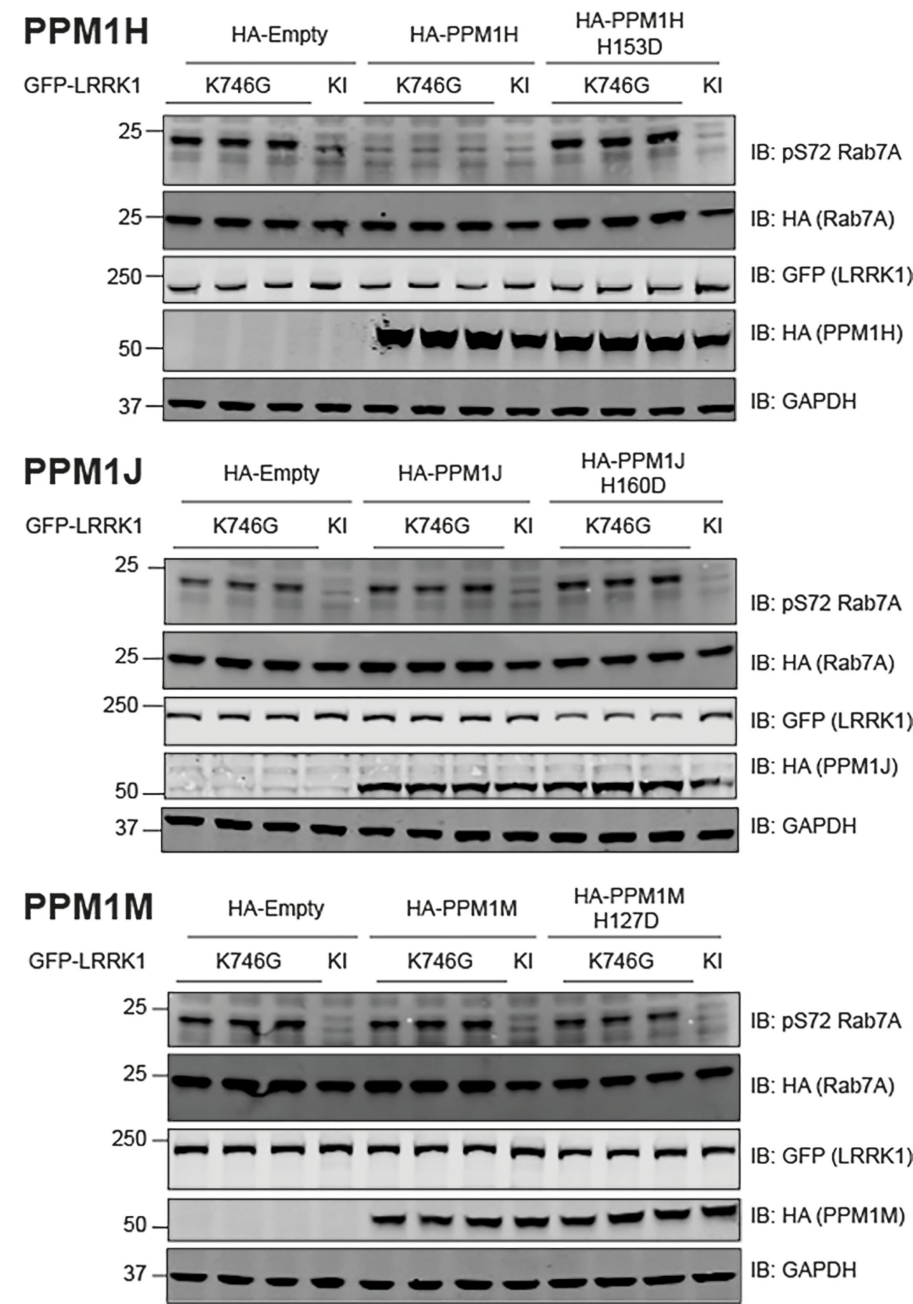
PP1MH, but not PPM1J or PPM1M, dephosphorylates Rab7A at Ser72. HEK293 cells were transiently transfected with the indicated plasmids encoding for wild type or catalytically inactive PPM1H (upper panel), PPM1J (middle panel) or PPM1M (lower panel), alongside LRRK1[K746G] or kinase inactive (KI) and wild type Rab7A. The kinase inactive (KI) mutant corresponds to LRRK1[D1409A]. Twenty-four hours post-transfection the cells were lysed and extracts (20 µg) from a duplicate experiment in which cells cultured in separate dishes were subjected to immunoblot analysis with the indicated antibodies (all at 1 µg/ml). Each lane represents cell extract obtained from a different replicate. The membranes were developed using the LI-COR Odyssey CLx Western Blot imaging system.

The raw data and requested correction have been assessed by and agreed with the Publisher. The authors apologise for the error and any inconvenience this may have caused. The data analysis and conclusions are not affected by the error.

The corrected Figure 9 is presented here.

