# Supplementary material for: Deciphering the LRRK code: LRRK1 and LRRK2 phosphorylate distinct Rab proteins and are regulated by diverse mechanisms
Source: Biochem J. 2021 Feb 10;478(3):553–78. doi: 10.1042/BCJ20200937 (PMC7886321; doi:10.1042/BCJ20200937)

Supplementary Figure S1: Coomassie gel of the purified recombinant LRRK1. 20 μl of the final gel filtration purification encompassing the peak of LRRK1 protein were analyzed by SDS-polyacrylamide electrophoresis and stained with Coomassie Blue. The fractions that were combined are indicated with a solid line.

Supplementary Figure S2. **PMA and calyculin A stimulates Rab7A phosphorylation in wild type MEFs**. Wild type MEFs were deprived of serum for 16 h and then incubated for 1 h in the presence or absence of the dose of indicated inhibitor. Cells were then stimulated with the dose of indicated agonists for the times indicated. Extracts (20 μg) from duplicate experiment were subjected to immunoblot analysis with the indicated antibodies (all at 1 µg/ml). Each lane represents cell extract obtained from a different cell dish. The membranes were developed using the LI-COR Odyssey CLx Western Blot imaging system.

Supplementary Figure S3. Rab7A phosphorylation induced by PMA is blocked by GO6983 PKC inhibitor. Wild type MEFs were deprived of serum for 16 h and then incubated for 1 h in the presence or absence of the dose of indicated inhibitor. Cells were then stimulated with PMA (100 ng/ml) for 30 min. Extracts (20 μg) from duplicate experiment were subjected to immunoblot analysis with the indicated antibodies (all at 1 µg/ml). Each lane represents cell extract obtained from a different cell dish. The membranes were developed using the LI-COR Odyssey CLx Western Blot imaging system.

Supplementary Figure S4. EGF-stimulation induces Rab7A phosphorylation at Ser72 by LRRK1 in HaCaT cells. (A) Wild type HaCat cells (human immortal keratinocytes) were deprived of serum for 16 h and then incubated for 1 h in the presence or absence of the dose of indicated inhibitor. Cells were then stimulated with the dose of indicated agonists for the times indicated. Extracts (20 μg) from duplicate experiment were subjected to immunoblot analysis with the indicated antibodies (all at 1 µg/ml). Each lane represents cell extract obtained from a different cell dish. The membranes were developed using the LI-COR Odyssey CLx Western Blot imaging system. (B) $2x10^{5}$ HaCaT cells/well were seeded in 6-well plates and after 24h transfected with 10nM of siRNAs against LRRK1, siRNA with scrambled sequence (scr) or left untransfected. After 60h, cells were serum starved overnight and then stimulated with EGF (50ng/ml) for 15min. Serum-starved, untransfected cells treated with Calyculin A (100nM, 5min) served as control. Immunoblots were developed using LI-COR Odyssey CLx Western Blot imaging system with the indicated antibodies at 1 µg/ml concentration.

Supplementary Figure S5. **TBK1 activation peaks after 20 mins of IL-1A treatment in wild type MEFs**. Wild type MEFs were deprived of serum for 16 h and then treated with IL-1A at the indicated time points. Extracts (20 μg) from duplicate experiments were subjected to immunoblot analysis with the indicated antibodies (all at 1 µg/ml). Each lane represents cell extract obtained from a different cell dish. The membranes were developed using the LI-COR Odyssey CLx Western Blot imaging system.

Supplementary Figure S6: The LRRK1[K746G+Y971F] double mutant does not further activate LRRK1. HEK293 cells were transiently transfected with the indicated plasmids encoding for wild type and mutant of LRRK1 and wild type Rab7A. Note that the Kinase inactive (KI) mutant corresponds to LRRK1[D1409A]. 24 h post-transfections the cells were lysed and extracts (20 μg) from a duplicate experiment in which cells cultured in separate dishes were subjected to immunoblot analysis with the indicated antibodies (all at 1 µg/ml). Each lane represents cell extract obtained from a different replicate. The membranes were developed using the LI-COR Odyssey CLx Western Blot imaging system

Supplementary Figure S7. **GSK2578215A, HG10-102-01, LRRK2-IN1 and iN04 do not inhibit LRRK1**. HEK293 cells were transiently transfected with the indicated plasmids

encoding for wild type Rab7A and the indicated wild type and mutant forms of LRRK1 or wild type LRRK2. 24 h post-transfections the cell were treated with the indicated concentrations of GSK2578215A (A), HG10-102-01 (B), LRRK2-IN1 (C) and iN04 (D) for 2 h. Cells were lysed and extracts (20 μg) from a duplicate experiment in which cells were cultured in separate dishes were subjected to immunoblot analysis with the indicated antibodies (all at 1 μg/ml). Each lane represents cell extract obtained from a different replicate. The membranes were developed using the LI-COR Odyssey CLx Western Blot imaging system. Similar results were obtained in 3 independent experiments.

Supplementary Figure S8. GZD-824 inhibits LRRK1 when overexpressed and *in vitro*. Quantification of GZD-824 dose-response experiments from Fig 6B (Upper) and 6C (Lower). Results are presented with log (Concentration of GZD-824, nM) on X-axis and pRab7A/total Rab7A relative to DMSO-treated control on Y-axis.

Supplementary Table 1. Single Nucleotide Polymorphisms in LRRK1.

**SNP ID**	**Allele**	**Residue Change**	**Mutation Type**	**Frequency**
rs766875506	G>T	E929*	Stop-gained	0.000008 (ExAC)
	G>A	E929K	Missense	
	G>C	E929Q	Missense	
rs947265550	A>G	Y971C	Missense	0.000012 (TOPMED)
rs1254240956	A>T	I1412F	Missense	0.000008 (TOPMED)
rs886038213	GAGTGGTdel	E1980A-FS*66	Frameshift	N/A [6]

Supplementary File 1.
The excel sheet contains the phospho Rab peptide sequences, the corresponding heavy and light m/z, their charge states and scheduled retention times used for targeted Mass spectrometry analysis of LRRK1 KO and LRRK1 wt MEFS.

Supplementary Figure S1

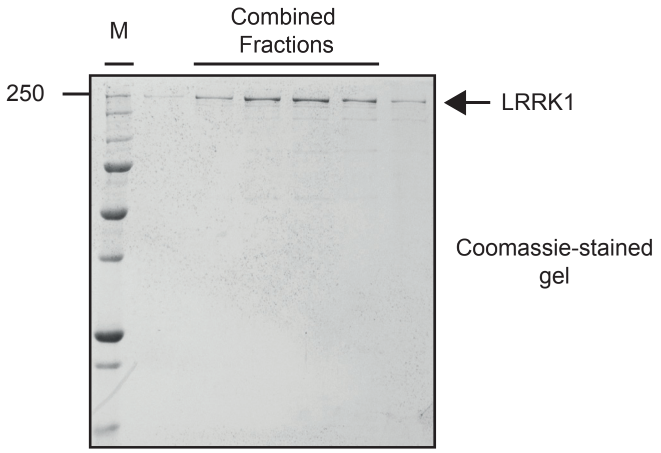

Supplementary Figure S2

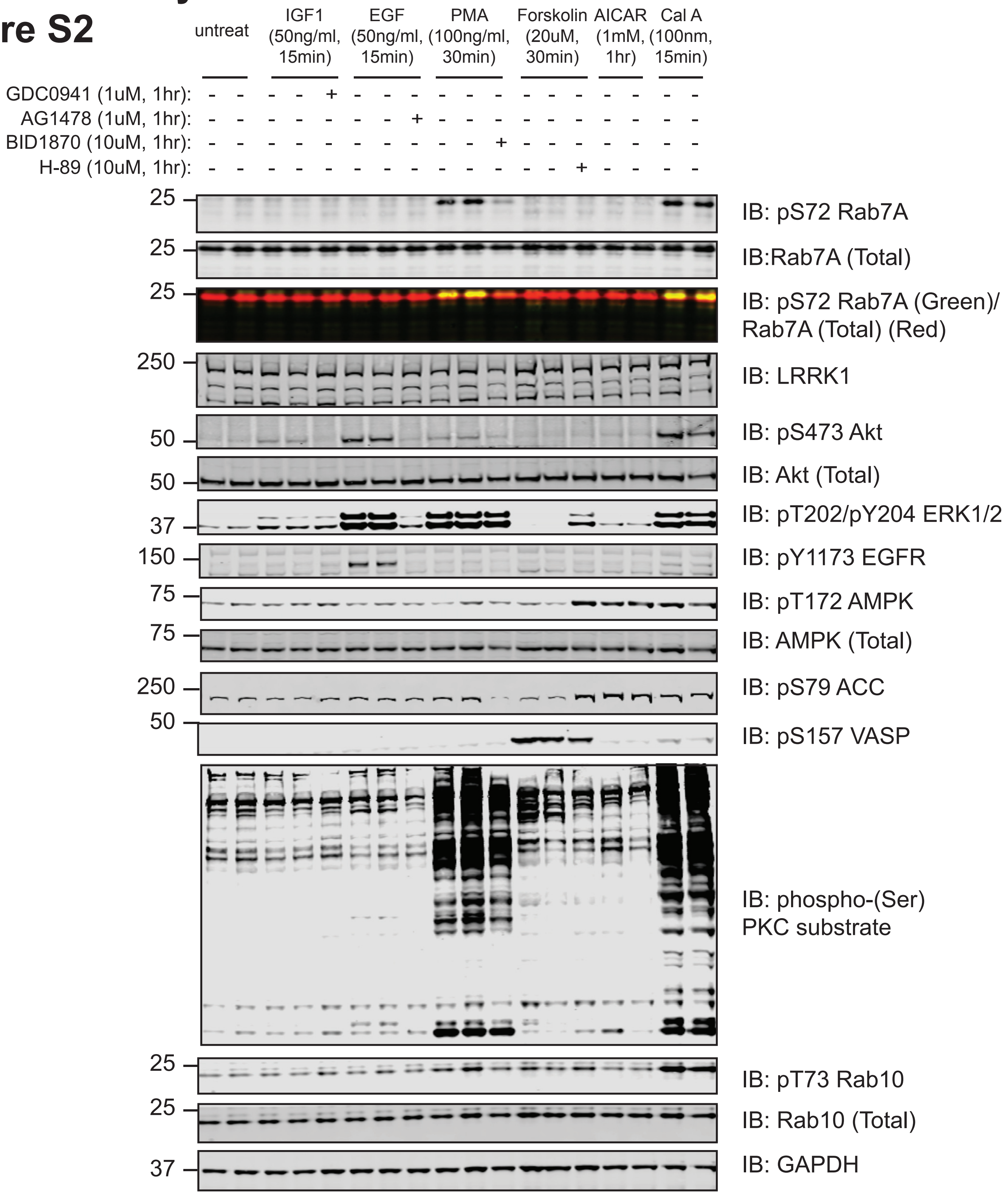

Supplementary Figure S3

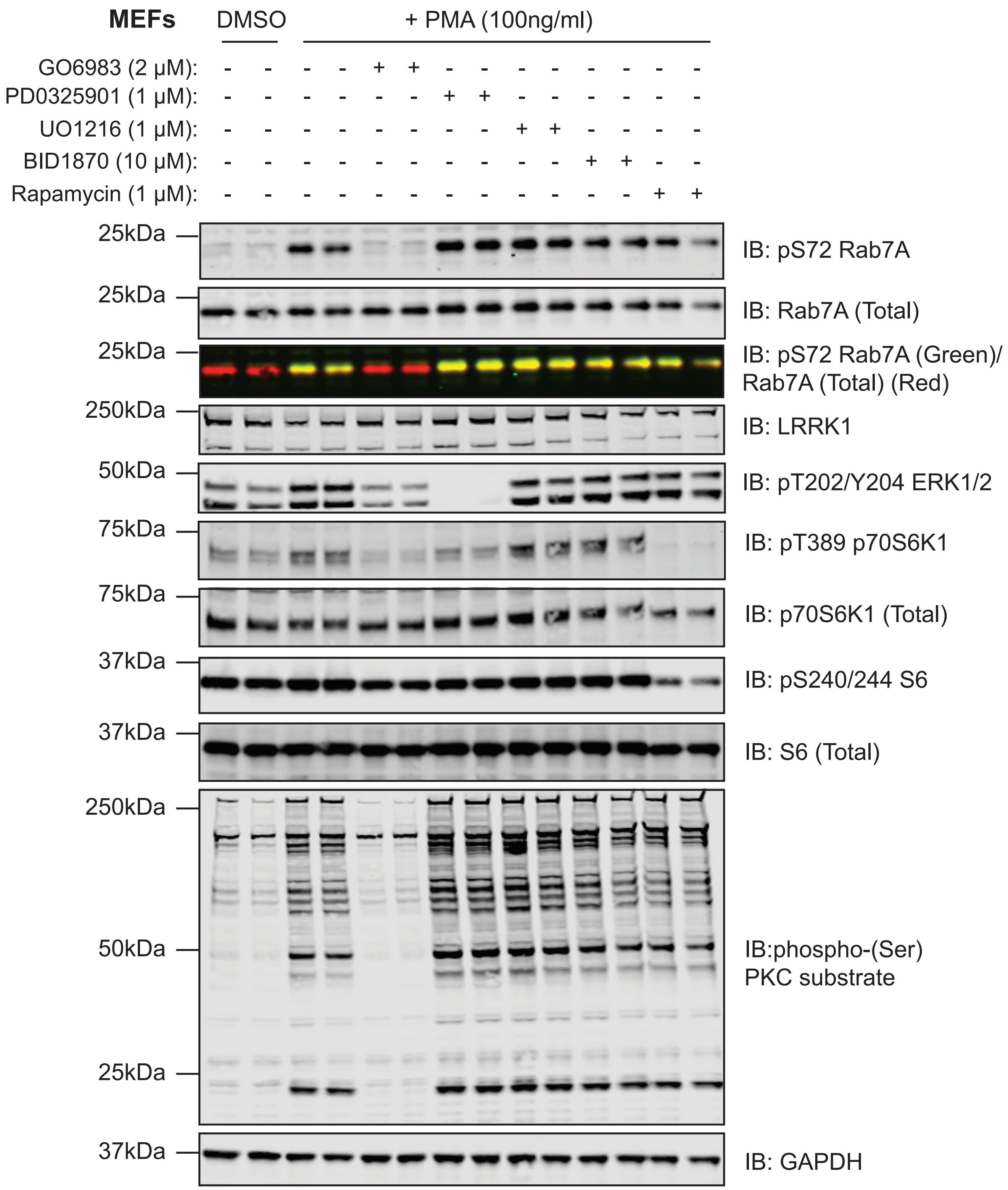

Supplementary Figure S4

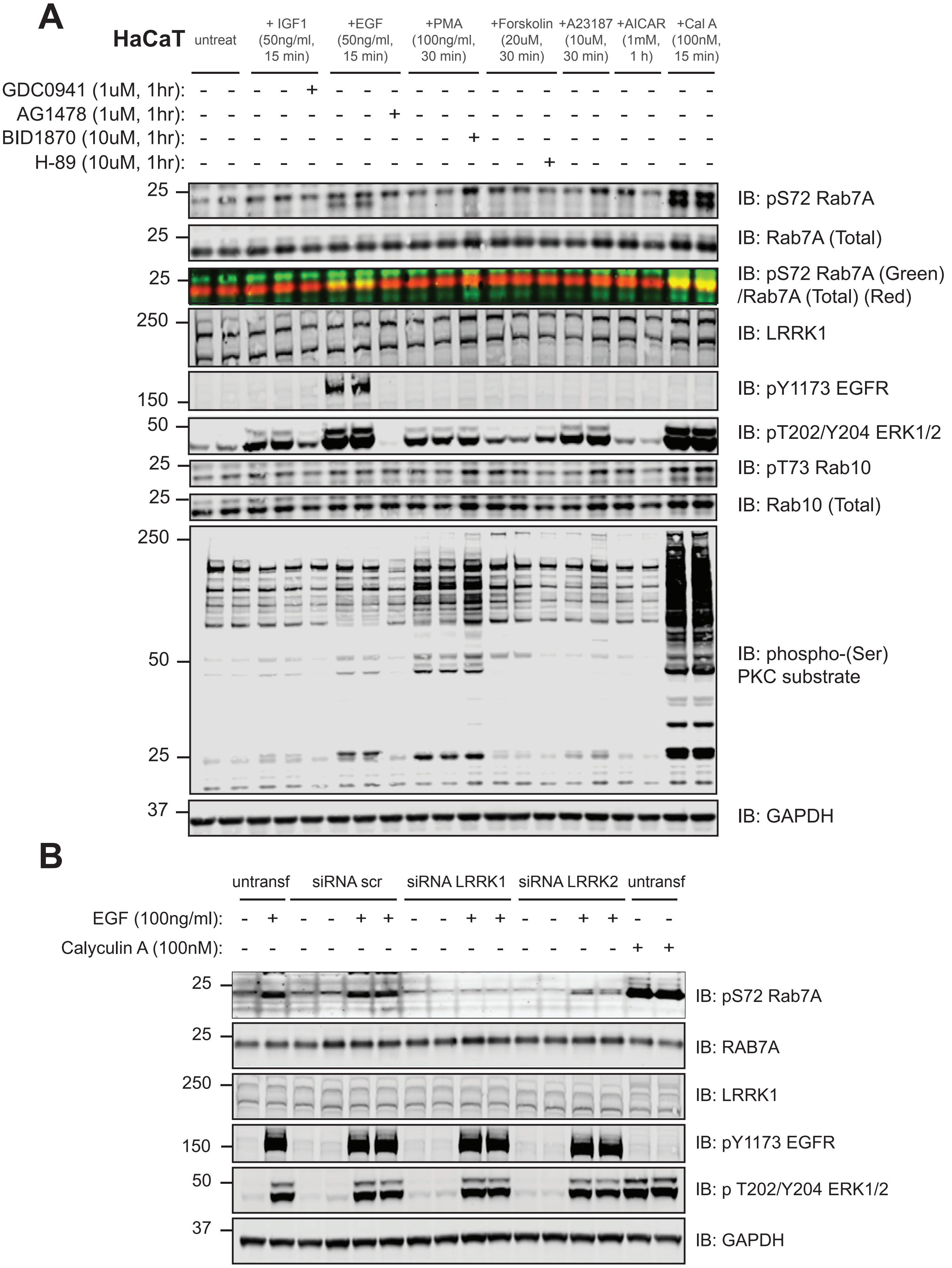

Supplementary Figure S5

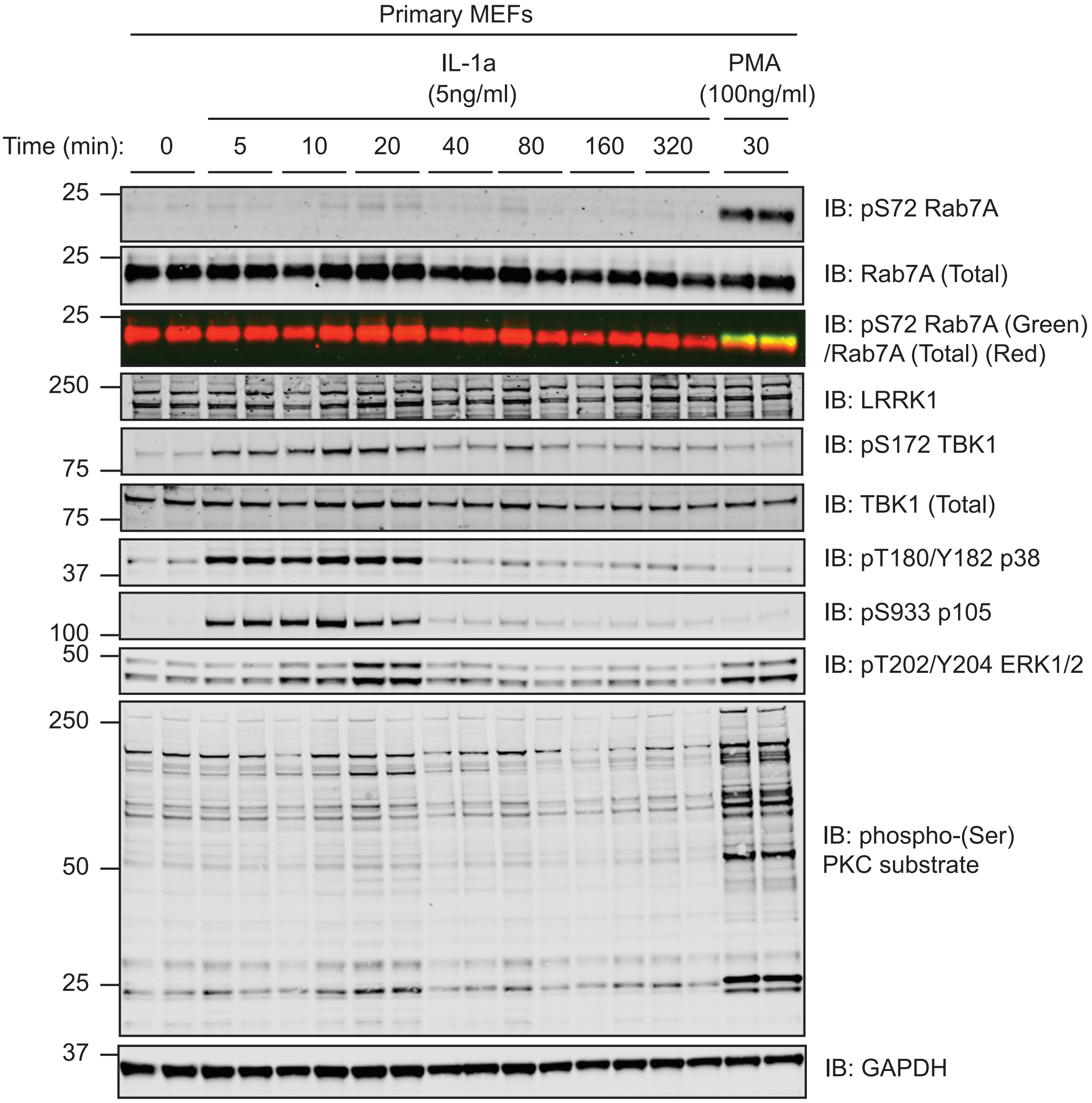

Supplementary Figure S6

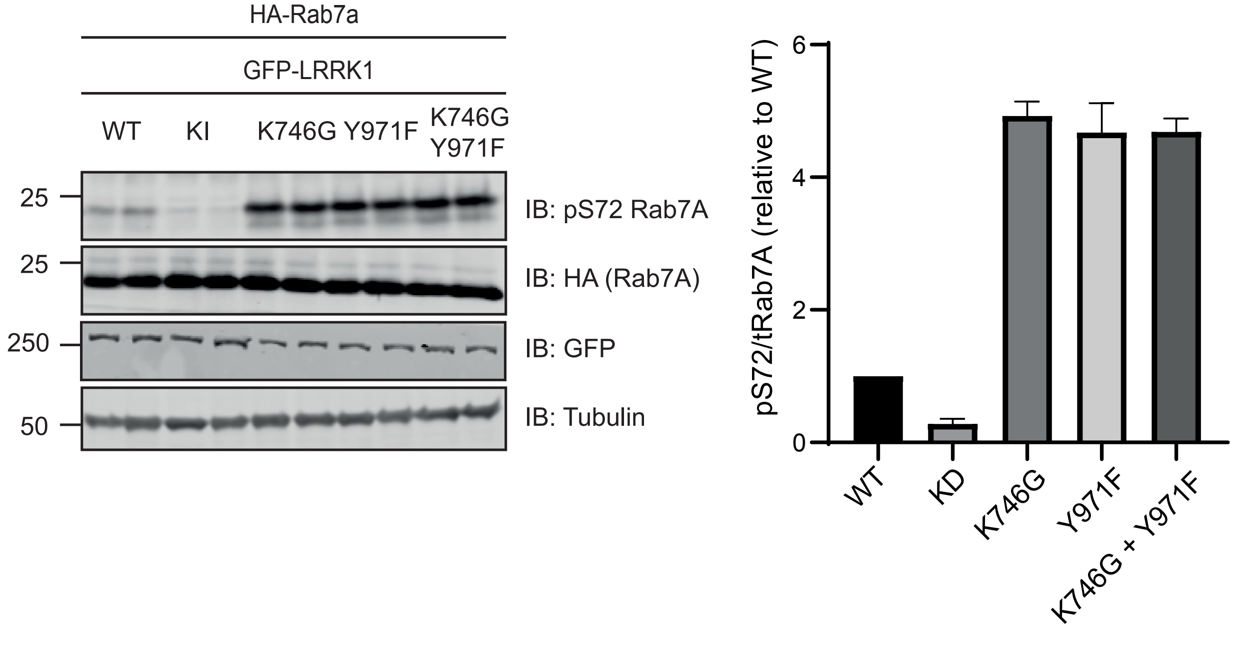

Supplementary Figure S7

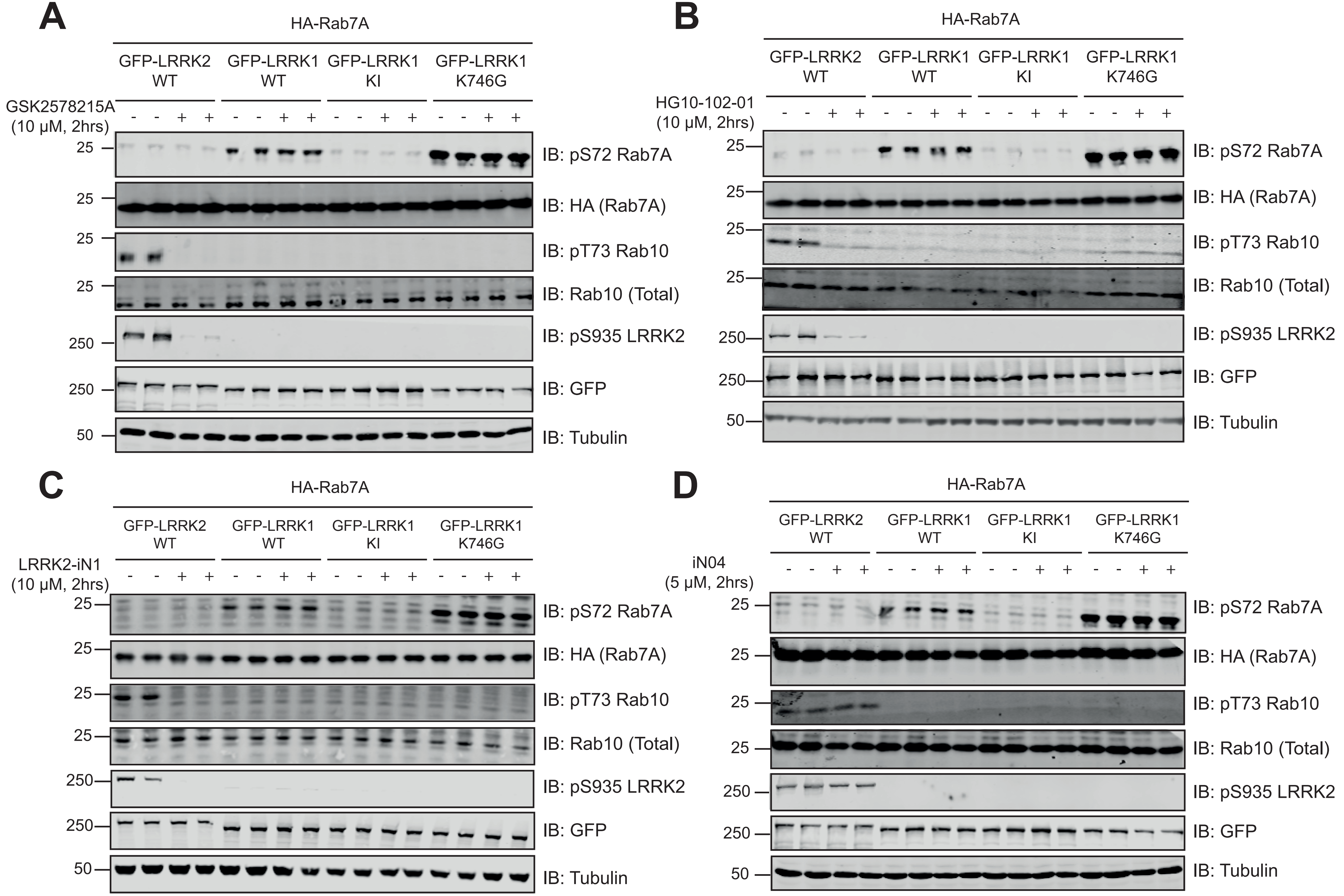

Supplementary Figure S8

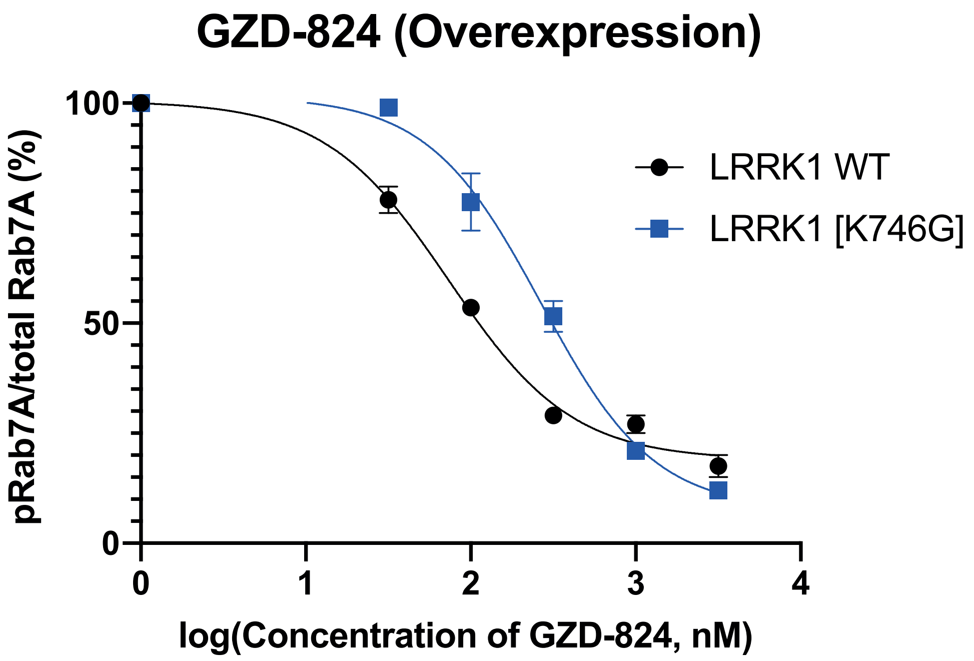

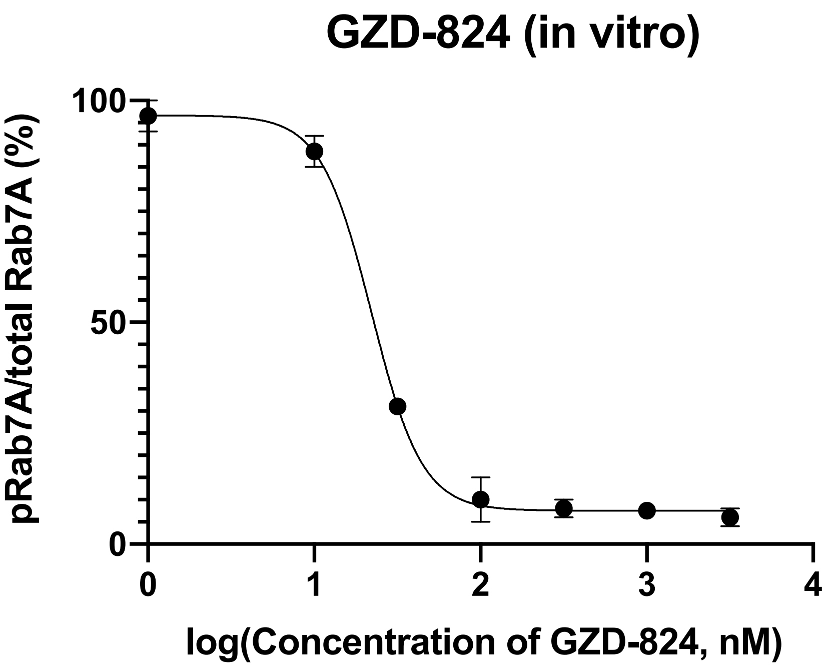

Supplement: Supplementary Figures S1-S8 and Table S1 [file BCJ-478-553-s1.pdf]
